# Current Approaches to the Management of Rheumatic Diseases in Pregnancy: Risk Stratification, Therapeutic Advances, and Maternal–Fetal Outcomes

**DOI:** 10.3390/jpm15090406

**Published:** 2025-09-01

**Authors:** Aikaterini-Gavriela Giannakaki, Maria-Nektaria Giannakaki, Anastasia Bothou, Konstantinos Nikolettos, Nikolaos Machairiotis, Kalliopi I. Pappa, Panagiotis Tsikouras

**Affiliations:** 1 First Department of Obstetrics and Gynecology, Alexandra University Hospital, National and Kapodistrian University of Athens, 11528 Athens, Greece; kpappa@med.uoa.gr; 2 Medical School, Aristotle University of Thessaloniki, 54124 Thessaloniki, Greece; manegiann@med.uoa.gr; 3 Department of Midwifery, University of West Attica, 12243 Athens, Greece; abothou@uniwa.gr; 4 Department of Gynaecological Oncology, Maidstone and Turnbridge Wells NHS Trust, Maidstone ME169QQ, UK; 5 Third Department of Obstetrics and Gynecology, University General Hospital “ATTIKON”, Medical School, National and Kapodistrian University of Athens, 12462 Athens, Greece; 6 Department of Obstetrics and Gynecology, Democritus University of Thrace, 68100 Alexandroupolis, Greece

**Keywords:** autoimmune rheumatic disease, pregnancy outcomes, systemic lupus erythematosus (SLE), antiphospholipid syndrome (APS), preconception counseling, maternal–fetal medicine, risk stratification

## Abstract

**Background:** Autoimmune rheumatic diseases, including systemic lupus erythematosus (SLE), antiphospholipid syndrome (APS), Sjögren’s syndrome, systemic sclerosis (SSc), and rheumatoid arthritis (RA), pose significant challenges during pregnancy and are associated with increased risks of adverse maternal and fetal outcomes, such as preeclampsia, fetal growth restriction (FGR), miscarriage, and preterm birth. The aim of this review is to synthesize recent evidence on pregnancy-related risks, preconception counseling, and therapeutic strategies for these conditions, with a particular focus on the importance of disease remission, pregnancy-compatible medications, and the selective use of biologics. **Methods:** A structured narrative review was conducted through a comprehensive PubMed search (2020–2025). Eligible studies addressed maternal–fetal outcomes, therapeutic approaches, and predictive factors in pregnant individuals with autoimmune rheumatic diseases. **Results:** Pregnancy outcomes have improved with early disease control and multidisciplinary care; however, major challenges persist. These include limited access to novel therapies, underrepresentation of diverse populations in clinical trials, and insufficient data on long-term neonatal outcomes. The strongest predictors of adverse outcomes remain disease activity at conception, specific autoantibody profiles, and systemic organ involvement. **Conclusions:** Optimal pregnancy outcomes for women with autoimmune rheumatic diseases require coordinated multidisciplinary care, the use of pregnancy-compatible medications, and achievement of prolonged disease remission prior to conception. Further research is needed to close existing knowledge gaps and ensure equitable, high-quality maternal–fetal care.

## 1. Introduction

Autoimmune rheumatic diseases, including systemic lupus erythematosus (SLE), antiphospholipid syndrome (APS), Sjögren’s syndrome, systemic sclerosis (SSc), and rheumatoid arthritis (RA), primarily affect women of reproductive age and frequently complicate pregnancy [1]. These disorders are associated with significantly increased risks of preeclampsia, fetal growth restriction (FGR), preterm birth, and pregnancy loss. Maternal outcomes are influenced by disease-specific autoantibodies, baseline organ involvement, and the use of immunosuppressive therapies [2].

Over the past two decades, advances in immunomodulatory therapies, risk stratification tools, and multidisciplinary care have led to improved maternal and neonatal outcomes [3]. However, active disease at conception and during pregnancy remains the strongest predictor of adverse outcomes.

Barriers to the universal implementation of best practices persist. These include healthcare inequities such as socioeconomic disparities, delayed diagnosis, and limited access to specialized rheumatological care [4]. Women from underprivileged or underserved populations are disproportionately affected, highlighting the complex interplay of systemic, clinical, and biological factors [5].

The aim of this review is to provide a comprehensive synthesis of the current evidence on preconception counseling, maternal–fetal outcomes, and therapeutic decision making for pregnant women with autoimmune rheumatic diseases. Furthermore, it identifies key research gaps, emphasizing the need for equitable, personalized, and multidisciplinary care for this high-risk population.

## 2. Methods and Materials

### 2.1. Study Design

This work is a structured narrative review synthesizing contemporary evidence on the management of rheumatic diseases during pregnancy. Although a systematic literature search was conducted, the review does not strictly follow PRISMA guidelines, as the aim was narrative synthesis and expert interpretation of findings.

### 2.2. Search Strategy

A comprehensive PubMed search was performed to identify relevant studies published between January 2020 and March 2025. The search focused on rheumatic diseases in pregnancy, including systemic lupus erythematosus (SLE), antiphospholipid syndrome (APS), Sjögren’s syndrome, systemic sclerosis (SSc), and rheumatoid arthritis (RA). The following MeSH terms and keywords were used, individually and in combination with “pregnancy”: Systemic Lupus Erythematosus, Antiphospholipid Syndrome, Rheumatoid Arthritis, Sjögren’s Syndrome, Systemic Sclerosis, Rheumatic Diseases. Boolean operators (AND and OR) were applied to refine the results. Only English-language studies involving human subjects were included. In addition, the reference lists of selected articles and recent meta-analyses were manually screened for additional relevant publications.

### 2.3. Study Selection

The initial search retrieved 1280 records, with an additional 40 studies identified through manual reference screening. After the removal of 270 duplicates, 1050 unique records were screened by title and abstract. Subsequently, 160 full-text articles were assessed for eligibility, and 95 studies met the inclusion criteria for the final synthesis.

### 2.4. Eligibility Criteria

Inclusion:

- Prospective and retrospective cohort studies;
- Randomized controlled trials (RCTs);
- Systematic reviews and meta-analyses.

Exclusion:

- Case reports or small case series;
- Non-peer-reviewed publications;
- Studies not addressing pregnancy-related outcomes or management strategies.

### 2.5. Data Extraction and Synthesis

Data on maternal and fetal outcomes, predictive risk factors, therapeutic strategies, and preconception counseling were extracted. Priority was given to high-quality studies, such as multicenter cohorts, randomized trials, and meta-analyses. No formal risk-of-bias assessment was performed, consistent with the narrative nature of this review. Discrepancies regarding study inclusion were resolved through consensus among the reviewers.

## 3. Results

### 3.1. Systemic Lupus Erythematosus (SLE) and Pregnancy

#### 3.1.1. Maternal and Fetal Outcomes

Systemic lupus erythematosus (SLE) is a chronic autoimmune disease that predominantly affects women of reproductive age, with a global prevalence of approximately 30–50 per 100,000 individuals and a female-to-male ratio of 9:1. Incidence varies geographically, ranging from 1 to 10 cases per 100,000 person-years, with higher rates in certain Asian and non-Caucasian populations [6,7,8].

Pregnancies in women with SLE are associated with increased risks of complications, including preterm delivery, fetal growth restriction (FGR), preeclampsia, miscarriage, and adverse maternal outcomes (AMOs), such as lupus flares and gestational diabetes mellitus (GDM) [6,7,8]. Women with SLE exhibit approximately 30% lower pregnancy rates compared to those of healthy controls [9].

Multicenter data from Japan revealed that fewer than half of all the pregnancies in women with SLE were planned, and the use of assisted reproductive technology (ART) was more than twice as frequent as that in the general population. The incidences of preterm birth (39.4%), preeclampsia (15.0%), and FGR (12.9%) were significantly elevated [10]. Similarly, a retrospective cohort study (*n* = 149 pregnancies in 98 women) found a live birth rate of 93.3%, yet 40% of the pregnancies were preterm, 35% had intrauterine growth restriction, and 4% resulted in miscarriage. Intrauterine fetal death occurred in 2.7%. Hypertension was significantly associated with miscarriage, preterm birth, preeclampsia, and lupus nephritis. The coexistence of antiphospholipid syndrome (APS) further increased the risks of preterm delivery, thrombotic events, and postpartum hemorrhage [11]. In pregnancies complicated by APS, lupus flare rates reached 40%, and adverse fetal outcomes were reported in up to 80% of the cases [12].

A Colombian cohort (*n* = 48 pregnancies in women with SLE) similarly demonstrated high rates of preterm birth (70.8%) and preeclampsia (52%), particularly among women with elevated SLEPDAI and SLICC scores or active lupus nephritis. Despite the presence of complications, including hypertensive disorders, eclampsia, and placental abruption, the fetal survival rate was 89.5% [13,14,15].

Lupus flares during pregnancy remain one of the most significant predictors of adverse outcomes [8,16,17]. Additional risk factors include advanced maternal age, coexisting APS, active lupus nephritis, corticosteroid use [6,7], disease activity at conception, hypocomplementemia, lupus anticoagulant positivity, and newly diagnosed SLE [8,16,17]. Neonatal outcomes closely mirror maternal disease activity. Although prematurity-related morbidities were not significantly different between infants born to mothers with and without SLE, leukocyte, neutrophil, and platelet counts were lower in neonates exposed to SLE, both at birth and during the first postnatal week [18]. Even in cases of clinically quiescent disease, pregnancy in women with SLE is considered as high risk and requires close monitoring and multidisciplinary care [16].

Notably, maintaining optimal maternal vitamin D levels (25(OH)D: 40–59 ng/mL) may help to reduce pregnancy-related complications in SLE [19].

#### 3.1.2. Preconception Risk Assessment

Achieving favorable pregnancy outcomes in women with SLE relies heavily on comprehensive preconception evaluation and optimal disease control. Pregnancies that occur prior to the clinical diagnosis of SLE have been linked to higher incidences of complications, such as preeclampsia and fetal growth restriction (FGR), suggesting that immune dysregulation may precede overt disease manifestations [20]. Conversely, another study reported higher rates of term delivery before an SLE diagnosis and identified aCL, IgG, and anti-β_2_ GPI IgG/IgM antibodies as markers associated with adverse outcomes [21]. A meta-analysis of 10,355 pregnancies in 8065 women with SLE demonstrated that lupus nephritis significantly reduces the probability of live birth (OR 0.62; 95% CI 0.47–0.81) and increases the risks of preterm delivery (OR 2.00; 95% CI 1.55–2.57) and preeclampsia (OR 3.11; 95% CI 2.35–4.12). Chronic hypertension was also associated with increased rates of disease flares, preterm birth, and preeclampsia. Secondary APS was linked to lower live birth rates and a higher incidence of pregnancy loss beyond 20 weeks of gestation [22]. Complement levels provide important prognostic information: Low preconception C4 levels predict gestational flares (OR 13.81, *p* < 0.001), and the absence of a physiological rise in C3/C4 levels during pregnancy is associated with adverse outcomes [23,24,25]. Established predictors of poor pregnancy outcomes include active disease; proteinuria; hypocomplementemia; lupus nephritis; aPL positivity; anti-Ro, anti-RNP, and anti-Sm antibodies; chronic hypertension; and a history of neuropsychiatric SLE. It is recommended that conception be postponed until the disease has remained inactive for at least six months [26,27].

#### 3.1.3. Pregnancy Management and Therapy

##### Screening and Baseline Interventions

Routine screening for antiphospholipid antibodies (LA, aCL, and anti-β_2_ GPI) is recommended, as their presence significantly increases the risk of pregnancy complications [28,29]. A multidisciplinary care approach improves maternal and fetal outcomes. A prospective study demonstrated an 86% live birth rate with coordinated care [30].

##### Medication Strategies

- Hydroxychloroquine (HCQ):

HCQ reduces lupus flares, prematurity, and preeclampsia [12,31]. It is recommended for all pregnant women with SLE, regardless of disease activity [32], and is considered as safe in pregnancy, although its impact on outcomes remains debated [33]. In a cohort from Karnataka (121 pregnancies in 80 women), HCQ use and remission at conception were associated with a flare rate of just 4.1%. Anti-Ro antibody positivity was also linked with reduced rates of preterm delivery [12,31]. While some findings did not reach statistical significance, women receiving HCQ during their first pregnancy showed a trend toward improved outcomes [34]. However, the RELESSER study (809 women and 1869 pregnancies) found that HCQ and steroids were not universally protective in high-risk pregnancies, while heparin was more commonly used in the context of APS [21]. Overall, HCQ may reduce disease activity and preeclampsia risk in SLE pregnancies but appears to be ineffective in patients with isolated aPL positivity or APS, and it does not significantly reduce the risk of intrauterine growth restriction [35].

- Glucocorticoids:

One study (*n* = 74 pregnancies) associated glucocorticoid doses of ≥10 mg/day with increased risk of preterm birth [36]. Conversely, other reports (149 pregnancies in 114 Thai women) suggested that prednisolone at >10 mg/day and the continuation of immunosuppressives at conception may protect against prematurity and flares [37].

- Immunosuppressive agents:

Discontinuation of immunosuppressives has been linked to disease flares in Japanese cohorts. Early declines in C3 were predictive of stillbirth, underscoring the importance of continuing immunosuppression and complement monitoring throughout pregnancy [38].

- Aspirin (ASA):

Low-dose aspirin (LDA) may reduce rates of very preterm birth and improve neonatal survival [18]. Some studies also showed reduced rates of preeclampsia, although not all the findings were statistically significant [39].

##### Special Considerations

In SLE pregnancies without renal involvement and with a negative aPL status, the baseline obstetric risk is relatively low [39]. A history of severe organ involvement has not been consistently associated with adverse pregnancy outcomes but may still correlate with increased risks of pregnancy loss, very preterm birth (<32 weeks), low birthweight (<1500 g), and congenital anomalies [40]. Despite extensive research, some cohorts have failed to identify reliable preconception predictors of adverse outcomes in SLE pregnancies [41], highlighting the multifactorial nature of risk and the need for individualized risk stratification.

### 3.2. Antiphospholipid Syndrome (APS) and Pregnancy

#### 3.2.1. Pathophysiology and Diagnostic Subtypes

Antiphospholipid syndrome (APS) is an autoimmune disorder characterized by the persistent presence of antibodies targeting phospholipids and their associated proteins, which contribute to both thrombotic events and pregnancy-related complications. It is considered as a rare condition, with an estimated annual incidence of 1–2 cases per 100,000 person-years and a prevalence of 40–50 per 100,000 individuals [42].

Obstetric APS (OAPS) is primarily mediated by antiphospholipid antibodies (aPL), particularly anti-β_2_-glycoprotein I antibodies. These autoantibodies impair placental development through non-thrombotic mechanisms, including inhibition of trophoblast invasion, defective spiral artery remodeling, and inflammation at the maternal–fetal interface.

Diagnosis is based on the revised Sydney classification criteria, which require clinical evidence of obstetric morbidity in combination with persistent laboratory positivity for aPL. However, a significant proportion of patients do not fulfill the full criteria:

- Obstetric morbidity APS (OMAPS): Clinical features consistent with APS in the absence of full laboratory confirmation;
- Non-criteria OAPS (NC-OAPS): Presence of laboratory markers without meeting the required clinical criteria.

Emerging evidence indicates that patients with NC-OAPS may achieve similar pregnancy outcomes to those with criteria-defined OAPS, provided they receive comparable treatment regimens [43,44].

Additionally, novel pathogenic mechanisms are increasingly recognized in the pathophysiology of OAPS. These include the involvement of extracellular vesicles, microRNAs, and neutrophil extracellular traps (NETs) [45]. Recent research has also identified intracellular signaling pathways in placental cells as direct targets of aPL. However, effective therapeutic strategies targeting these molecular pathways remain under investigation and are yet to be validated in clinical practice [46].

#### 3.2.2. Maternal and Fetal Outcomes

Antiphospholipid antibodies (aPL) have been associated with a spectrum of adverse pregnancy outcomes:

- Fetal Growth Restriction (FGR): Strongly associated with anticardiolipin (aCL) and anti-β_2_-glycoprotein I (anti-β_2_ GPI) antibodies but not significantly linked with lupus anticoagulant (LA) positivity [47];
- Preterm Birth: More commonly observed in women with multiple aPL positivity, particularly those with triple positivity [48];
- Stillbirth at (≥10 weeks): APS was identified as the underlying etiology in approximately 10.3% of cases, suggesting possible underdiagnosis. This underscores the need for repeat serological testing and extended postpartum follow-up in affected women [49];
- Severe Thrombocytopenia (at <50 × 10^9^/L): Associated with heightened risks of preterm delivery, uteroplacental insufficiency, and small-for-gestational-age (SGA) neonates [50].

A comprehensive meta-analysis identified several key predictors of adverse pregnancy outcomes in APS. Triple aPL positivity was associated with significantly lower odds of live birth (OR = 0.33) and higher odds of preeclampsia (OR = 2.43) and SGA neonates (OR = 2.47). LA positivity was linked with increased risks of preeclampsia (OR = 2.10), SGA (OR = 1.78), and preterm birth (OR = 3.56). Prior thrombotic events were strongly predictive of neonatal death (OR = 15.19) and SGA (OR = 2.60), emphasizing their critical prognostic value [51].

#### 3.2.3. Preconception Risk Assessment

Anti-β_2_ GPI/HLA-DR antibodies may define a distinct subset of women with recurrent pregnancy loss, offering both diagnostic utility in seronegative APS and potential targets for novel therapies [52]. Moreover, even low titers of aCL antibodies, particularly IgG isotypes, have been associated with increased pregnancy risk, supporting the rationale for first-trimester aCL screening [53]. A high-risk maternal profile is characterized by lupus anticoagulant (LA) positivity, evidence of placental dysfunction, coexisting hypertension, previous preterm delivery at or before 34 weeks, and persistent aPL positivity. These patients warrant early risk stratification, intensified surveillance, and personalized treatment strategies to mitigate adverse outcomes [54].

#### 3.2.4. Fetal Surveillance and Predictive Modeling

A prospective cohort study of 105 pregnant women with OAPS demonstrated alterations in fetal cardiac function, including increased myocardial performance index (MPI) and isovolumetric relaxation time (IVRT), as well as reduced ejection time (ET), middle cerebral artery pulsatility index (MCA-PI), and cerebroplacental ratio (CPR) compared to those of healthy controls. Multivariate analysis identified gravidity, MPI, and CPR as independent predictors of adverse pregnancy outcomes (APOs). A clinical nomogram based on these parameters showed high predictive accuracy (AUC = 0.923) [55]. Additional studies have highlighted the prognostic value of anti-β_2_ GPI domain 1 (aD1) and antiphosphatidylserine/prothrombin (aPS/PT) IgG antibodies, which were independently associated with APOs, reflecting distinct pathogenic mechanisms involving placental insufficiency and fetal demise [56].

#### 3.2.5. Treatment Strategies

##### Standard Therapy

The combination of low-dose aspirin (LDA) and heparin, either low-molecular-weight heparin (LMWH) or unfractionated heparin (UFH), significantly improves pregnancy outcomes in women with APS-related recurrent pregnancy loss. One study reported a live birth rate of 87.2% in treated patients compared to 50.0% in those without anticoagulant treatment [52]. In contrast, aspirin monotherapy was associated with higher rates of preterm birth and lower neonatal birthweights [57].

##### Adjunctive Therapy

- Hydroxychloroquine (HCQ):

Although the role of HCQ in APS remains under investigation [58], it may offer therapeutic benefit in refractory OAPS by modulating non-thrombotic mechanisms [59,60]. When added to standard therapy with LDA and enoxaparin, HCQ improved live birth rates and reduced the frequency of pregnancy complications [61].

- Low-dose prednisone:

Low-dose glucocorticoids have shown benefit in refractory OAPS. In multivariate analyses, prednisone was the only intervention independently associated with improved pregnancy outcomes [62].

- Intravenous immunoglobulin (IVIg):

IVIg may be helpful in women with a history of stillbirth or treatment-resistant OAPS. When combined with LDA, LMWH, HCQ, and low-dose prednisone, IVIg has been associated with successful pregnancy outcomes in several observational cohorts [63,64].

##### Comprehensive Regimens

Intensified treatment strategies, such as HCQ + IVIg + plasma exchange (PEX) or immunoadsorption (IA), in combination with pravastatin and low-dose steroids, have achieved 100% live birth rates in select high-risk populations [65]. However, some regimens were also associated with elevated rates of maternal or fetal complications, emphasizing the importance of individualized treatment planning.

##### Evidence Synthesis

A systematic review concluded that no single therapeutic regimen consistently prevents fetal growth restriction (FGR), due to heterogeneity in study designs and overall low-quality evidence [63]. Nonetheless, both aspirin monotherapy and no treatment were linked to higher risks of fetal and neonatal deaths, while regimens including aspirin + corticosteroids or IVIg were more frequently associated with preterm delivery. For refractory APS, preconception initiation of low-dose IVIg (<2 g/kg/month) or HCQ at 400 mg/day may offer meaningful clinical benefit [66].

### 3.3. Sjogren’s Disease and Pregnancy

#### 3.3.1. Maternal and Fetal Outcomes

Mostly affecting women of reproductive age, Sjögren’s syndrome (SS) is an autoimmune disease characterized by systemic manifestations and lymphocytic infiltration of the exocrine glands. Recent epidemiological data estimate an annual incidence of approximately 6.9 per 100,000 person-years and a point prevalence of 60.8 per 100,000, with female-to-male ratios ranging from 9:1 to 28:1 [67].

A meta-analysis reported significantly elevated risks of adverse pregnancy outcomes in women with SS, including spontaneous abortion (OR = 9.30), preterm birth (OR = 2.19), low birthweight (OR = 3.84), and congenital anomalies (OR = 4.38) [67]. A large population-based study further confirmed increased odds of hypertensive disorders during pregnancy (gestational hypertension: OR = 1.65; preeclampsia/eclampsia: OR = 2.06), along with cesarean delivery and serious maternal complications, such as thromboembolism [68].

Fetal outcomes were similarly affected, with higher rates of spontaneous abortion, preterm birth, small-for-gestational-age (SGA) infants, and low birthweight (<2500 g). Notably, live birth rates were significantly reduced in SS pregnancies (OR = 21.53), and cesarean delivery was more common across all ethnic groups [68]. Among fetal complications, congenital heart block (CHB) is the most severe, associated with maternal anti-Ro/SSA and anti-La/SSB antibodies. Also, CHB can develop even in asymptomatic mothers, necessitating close fetal cardiac surveillance [68]. Other adverse outcomes include stillbirth, prematurity, and neonatal lupus, particularly in cases of active or poorly controlled disease [67,68]. Although neonatal lupus is rare, it may result from the transplacental passage of maternal autoantibodies, presenting with rash, cytopenias, or hepatic involvement. Subclinical cardiac involvement is possible, further supporting the use of serial fetal echocardiography between 16- and 28-weeks’ gestation [68]. Evidence suggests that women with primary SS (pSS) who receive adequate prenatal care may experience favorable outcomes. However, the presence of additional autoantibodies, such as antiphospholipid antibodies (aPL), further increases the risks of preterm birth and intrauterine growth restriction [69,70]. From the maternal perspective, SS usually remains clinically stable during pregnancy, although fatigue, mucosal dryness, systemic symptoms, and infection risk may worsen, complicating immunosuppressive management. SS has also been associated with increased odds of pregnancy-induced hypertension (OR = 1.65), preeclampsia (OR = 2.06), cesarean delivery (OR = 2.07), and thromboembolic events (OR = 9.45) [68].

#### 3.3.2. Preconception Risk Assessment

Preconception assessment in women with SS should include serological testing for anti-Ro/SSA and anti-La/SSB antibodies, given their strong associations with fetal cardiac manifestations, particularly congenital heart block (CHB) [68]. Identifying high-risk serological profiles enables individualized counseling, guides the intensity of fetal monitoring, and informs delivery planning.

#### 3.3.3. Treatment Strategies

Pregnancy management in women with SS must be individualized. Although the disease often remains clinically stable, anti-Ro/SSA and anti-La/SSB-positive women require enhanced fetal surveillance. Weekly fetal echocardiography from 16 to 28 weeks’ gestation is indicated to detect early signs of CHB, including persistent fetal bradycardia (<110 bpm), a prolonged PR interval (>150 ms), and ventricular dysfunction [68].

Recent evidence has suggested that maternal anti-Ro52 and anti-Ro60 titers below 110 AU/mL (via multiplex assay) or 50 U/mL (via ELISA) are associated with a negligible risk of CHB. In women without previously affected offspring, such low titers provide a 100% negative predictive value for CHB, supporting a risk-adapted approach to fetal monitoring [71].

Hydroxychloroquine (HCQ) is recommended preconceptionally and throughout pregnancy in anti-Ro/SSA-positive women, as it may reduce the risk of fetal cardiac complications [70]. Corticosteroids and immunosuppressants should be reserved for clearly indicated cases; prophylactic use is discouraged due to potential risks.

Optimal care requires close collaboration among rheumatologists, maternal–fetal medicine specialists, and pediatric cardiologists to ensure favorable outcomes for both mother and child [68].

### 3.4. Systemic Sclerosis and Pregnancy

#### 3.4.1. Maternal and Fetal Outcomes

Systemic sclerosis (SSc) is a rare autoimmune connective tissue disease, with a global incidence of approximately 1.4 per 100,000 person-years (95% CI: 1.1–1.9) and a prevalence of about 17.6 per 100,000 individuals (95% CI: 15.1–20.5). The disease exhibits a strong female predominance, with women affected five times more often than men [72].

Pregnancy in women with SSc is associated with significantly increased maternal and fetal risks, particularly when conception occurs after the diagnosis of the disease. A study of 60 pregnancies in women with SSc reported that only about 60% of post-diagnosis pregnancies resulted in live births, while rates of spontaneous abortion (20%), preterm delivery (26.7%), and low birthweight (20%) were substantially elevated [73]. Although the overall rate of pregnancy loss is not dramatically higher than that in the general population, women with diffuse cutaneous SSc or major organ involvement face particularly high risks. Preterm birth is a common complication across autoimmune diseases, including SSc. The most favorable pregnancy outcomes are seen in women with limited cutaneous SSc and stable disease, especially when managed with close monitoring. A multidisciplinary approach, involving rheumatologists, obstetricians, and maternal–fetal medicine specialists, is crucial for effective risk mitigation and management [74]. In a case-control study involving 187 women, more than 70% of those who were later diagnosed with SSc had previously experienced obstetric complications, including hypertensive disorders, preterm birth, and maternal infections, compared to 50% of matched controls [75]. This finding suggests potential early subclinical autoimmune activity prior to overt disease onset. Furthermore, active disease during pregnancy is associated with significantly worse outcomes, while pregnancies occurring during periods of disease quiescence, particularly under specialist supervision, are generally associated with favorable maternal and neonatal outcomes [76].

#### 3.4.2. Preconception Counseling

All women with systemic sclerosis (SSc) should receive comprehensive preconception counseling. Those with severe internal organ involvement, such as pulmonary arterial hypertension (PAH), a history of scleroderma renal crisis, or advanced interstitial lung disease (ILD), are considered as being at high risk, and pregnancy may be contraindicated due to the potential for life-threatening maternal and fetal complications. These women should receive individualized counseling regarding potential outcomes and be informed of alternative reproductive options [74].

For women with limited cutaneous SSc and stable disease, pregnancy is generally feasible following a thorough preconception evaluation, which should include:

- Cardiopulmonary assessment (e.g., echocardiography and pulmonary function tests);
- Renal function evaluation;
- Autoantibody screening, including anti-Scl-70 and anti-RNA polymerase III;
- Screening for subclinical pulmonary hypertension.

A disease-free period of at least six months is advised before conception to reduce the risks of maternal and fetal complications. Teratogenic agents, such as mycophenolate mofetil, methotrexate, and cyclophosphamide, must be discontinued at least 3–6 months prior to planned conception [77,78].

#### 3.4.3. Treatment Strategies

Teratogenic immunosuppressive or vasoactive agents should be replaced with pregnancy-compatible therapies, selected according to disease severity and organ involvement. Recommended management includes:

- Nifedipine or other calcium channel blockers: First-line agents for Raynaud’s phenomenon and digital ulcers, considered as safe in pregnancy [74];
- Low-dose aspirin: Frequently used to lower the risk of preeclampsia, particularly in women with vasculopathy or aPL positivity [74].

Immunosuppressive and anti-inflammatory agents with favorable pregnancy safety profiles:

- Hydroxychloroquine;
- Azathioprine;
- Sulfasalazine (with folic acid supplementation);
- Selected TNF inhibitors, such as certolizumab pegol (which has minimal placental transfer and can be continued throughout pregnancy) and etanercept (which may also be used in selected cases).

Other biological agents, including infliximab and adalimumab, are typically discontinued during the third trimester to minimize interference with infant vaccination schedules [78].

### 3.5. Rheumatoid Arthritis and Pregnancy

#### 3.5.1. Maternal and Fetal Outcomes

Rheumatoid arthritis (RA) is a common systemic autoimmune disease affecting approximately 0.5–1% of the global adult population. As of 2021, the global age-standardized prevalence rate reached 208.8 per 100,000 individuals, with an incidence of 11.8 per 100,000 person-years. Women are affected approximately 2.5 times more frequently than men [79].

Pregnancy in women with RA is associated with increased risks of both maternal and fetal complications. Preterm birth occurs in about 27.5% of RA pregnancies—markedly higher than the 5.6% rate observed in the general population [10]. Importantly, the soluble-fms-like tyrosine kinase-1 (sFlt-1)/placental growth factor (PlGF) ratio, a widely used biomarker for preeclampsia, remains unaffected by RA disease activity or sulfasalazine use, and a threshold value of ≤38 retains its predictive value in this population [80]. A meta-analysis of 18 observational studies involving more than 50 million individuals reported that maternal RA is associated with increased odds of cesarean section (OR = 1.39), preeclampsia (OR = 1.48), gestational hypertension (OR = 1.34), and spontaneous abortion (OR = 1.16) [81]. Fetal outcomes were also adversely affected, with higher rates of preterm birth, small-for-gestational-age (SGA) infants, low birthweight, congenital anomalies, and stillbirth [82,83]. Additionally, a Mendelian randomization analysis using GWAS data demonstrated that genetically predicted RA is associated with a modestly increased risk of preeclampsia (*p* < 0.05), a finding supported by multiple complementary statistical models [84,85]. While some studies suggest a potential link between maternal RA and neurodevelopmental disorders in offspring, current evidence remains insufficient to establish causality [86].

#### 3.5.2. Preconception Counseling

Preconception counseling in RA should include comprehensive medication review, optimization of disease activity, and coordination between rheumatology and obstetrics teams. Women are advised to conceive during periods of low or stable disease activity, under pregnancy-compatible therapies, and following a treat-to-target approach. Additional considerations include subfertility, autoantibody status (e.g., rheumatoid factor and anti-CCP), and musculoskeletal limitations, which may impact labor positioning, delivery, and anesthetic management. Given the elevated risks of hypertensive disorders, preeclampsia, and fetal growth restriction, early risk stratification and individualized monitoring protocols are recommended to improve maternal and fetal outcomes [87].

#### 3.5.3. Treatment Strategies

Medications compatible with pregnancy include:

- Hydroxychloroquine;
- Sulfasalazine;
- Azathioprine;
- Cyclosporine;
- Tacrolimus;
- Selected TNF inhibitors (e.g., adalimumab and etanercept).

Teratogenic agents, such as methotrexate, leflunomide, mycophenolate mofetil, and cyclophosphamide, must be discontinued prior to conception, due to the risk of fetal harm. In cases where conventional disease-modifying antirheumatic drugs (DMARDs) are insufficient, biological agents may be considered under specialist supervision, including TNF inhibitors (e.g., adalimumab and etanercept), IL-6 receptor blockers (e.g., tocilizumab) and B-cell-depleting agents (e.g., rituximab) [88,89].

Emerging evidence suggests that when clinically justified, biologics, especially TNF inhibitors, can be used safely during pregnancy, with no significant increase in adverse maternal or neonatal outcomes [90]. Furthermore, standard infant vaccination schedules remain appropriate following in utero exposure to anti-TNFα therapies. Currently, no strong evidence links non-TNF biologics or targeted synthetic DMARDs to poor pregnancy outcomes, although additional prospective studies are warranted to confirm their safety profiles [91]. Many women with RA experience spontaneous improvement in symptoms during pregnancy, though the mechanisms remain incompletely understood. A systematic review of 37 original studies identified multiple immunological changes that may underlie this modulation, including increased IgG galactosylation, shifts in pro- and anti-inflammatory cytokine levels, maternal–fetal HLA class II incompatibility, and gene expression alterations in immune cells. Other contributing factors include pregnancy-associated α2-glycoprotein, expansion of regulatory T-cells, and fetal microchimerism. Despite these promising leads, findings across studies are heterogeneous and, at times, conflicting, underscoring the need for standardized research protocols and longitudinal investigations to clarify the immunological pathways that contribute to RA amelioration during pregnancy [92]. A comparative overview of the clinical features, pregnancy risks, and therapeutic strategies across autoimmune rheumatic diseases is presented in Table 1.

## 4. Discussion

Pregnancy in women with autoimmune rheumatic diseases presents a complex clinical scenario that demands meticulous planning, individualized care, and interdisciplinary collaboration. The heterogeneity of disease presentations, overlapping serological profiles, and the need to balance disease control with fetal safety render management particularly challenging. Although each condition, SLE, APS, RA, SSc, and Sjögren’s syndrome, carries unique risks, the unifying theme across these disorders is the importance of maintaining disease quiescence before and during pregnancy. This discussion integrates findings from the reviewed evidence and explores the broader implications for clinical practice, healthcare systems, and research directions.

Clinical Interpretation of Findings

This comprehensive review highlights that active disease at the time of conception is the most consistent and critical predictor of adverse pregnancy outcomes in women with autoimmune rheumatic diseases. In SLE, active nephritis, and hypocomplementemia, elevated anti-Ro/La antibodies are associated with fetal loss, preterm birth, and hypertensive disorders. In APS, triple aPL positivity or prior thrombosis signals high risk for obstetric complications, including stillbirth, preeclampsia, and intrauterine growth restriction. Similarly, RA, SSc, and Sjögren’s syndrome are associated with increased rates of preterm birth, cesarean delivery, and congenital anomalies, particularly in the context of poor disease control or the presence of specific autoantibodies. Organ involvement and serological profiles should guide risk stratification. For example, complement levels in SLE, aPL profiles in APS, and anti-Ro titers in Sjögren’s syndrome have strong predictive values. The role of multidisciplinary management is paramount: Rheumatologists, maternal–fetal medicine specialists, and cardiologists must coordinate closely to implement surveillance strategies, such as serial fetal echocardiography and Doppler studies. Personalized care protocols tailored to disease severity and serological risk factors enhance maternal and neonatal safety.

Therapeutic Considerations and Medication Safety

The management of rheumatic diseases during pregnancy requires careful selection of medications to balance maternal disease control with fetal safety. Current international guidelines [76,93,94,95,96,97] recommend the use of several agents considered as safe during pregnancy, while others should be avoided due to teratogenicity or fetal toxicity. Table 2 summarizes the most commonly used medications, their recommended doses, and their pregnancy safety profiles.

Implications for Clinical Practice

Standardizing preconception counseling and individualized reproductive planning is essential for all women of childbearing potential with autoimmune rheumatic diseases. Preconception visits should include thorough evaluation of disease activity, assessment of high-risk serological markers, medication reconciliation, and patient education. Teratogenic agents, such as methotrexate, mycophenolate mofetil, and cyclophosphamide, should be withdrawn or substituted at least 3–6 months before attempting conception.

A treat-to-target approach using pregnancy-compatible DMARDs, with routine monitoring and prompt responses to disease flares, has shown to improve outcomes. Multidisciplinary care models significantly reduce maternal and fetal complications and improve patient satisfaction. These should be implemented universally and extended into the postpartum period, which remains a vulnerable time for disease exacerbation, thrombotic events, and the need for therapeutic adjustment. Integrated care pathways and follow-up extending into the neonatal period ensure continuity and safety in maternal and infant health [98].

Research Gaps and Health Equity

Despite encouraging advancements, critical gaps persist in our understanding of how immunological, pharmacological, and sociodemographic factors converge to influence outcomes. The current literature is dominated by observational studies, many of which lack standardized disease activity metrics and sufficient representation of diverse ethnic and racial populations. Minority women, particularly those identifying as Black, continue to experience disproportionately high rates of adverse maternal and fetal outcomes in diseases like SLE and APS.

These disparities are not solely biologically mediated but stem from structural inequities, such as delayed diagnosis, reduced access to subspecialty care, and systemic healthcare bias. Future research must prioritize equity-focused methodologies, including stratified analyses by race and ethnicity, and include diverse patient populations in clinical trials. Policy-driven incentives for inclusive recruitment and standardized outcome reporting can further address these inequities. Health equity should be embedded as a central metric in future clinical trials and observational registries [99].

Emerging Tools and Future Perspectives

The future of autoimmune pregnancy management lies in the integration of precision medicine and digital health innovations. Genomic, transcriptomic, and proteomic analyses can stratify patients based on molecular risk profiles, while artificial intelligence and machine-learning tools may help to refine predictive models for obstetric outcomes. These technologies hold promise in identifying patients at the highest risk, tailoring surveillance protocols, and guiding therapeutic interventions.

Furthermore, the creation of global pregnancy registries and the gathering of empirical data will be crucial for assessing the long-term safety of targeted and biological treatments administered during pregnancy. Policymakers must invest in developing high-risk pregnancy centers, ensure equitable access to necessary medications, and support ongoing provider education in autoimmune disease and pregnancy management. Translating scientific progress into practical benefit requires robust healthcare systems that foster multidisciplinary patient-centered care pathways and remove systemic barriers to care [1].

## 5. Conclusions

Autoimmune rheumatic diseases complicate pregnancy through a variety of immunological and clinical mechanisms. However, adverse pregnancy outcomes have significantly improved due to better disease control, compatible therapies, and collaborative care models. Achieving disease remission before conception, using medications with established safety profiles during pregnancy, and ensuring multidisciplinary management are fundamental steps to optimize maternal and fetal outcomes. Ongoing research efforts and systemic healthcare support remain essential to address persistent gaps and to provide equitable, high-quality care for this vulnerable population.

## Figures and Tables

**Table 1:** Overview of pregnancy management considerations in major autoimmune rheumatic diseases.

Disease	High-Risk Features	Recommended Therapies	Monitoring Focus	Main Pregnancy Risks
Systemic Lupus Erythematosus (SLE)	Active nephritis, hypocomplementemia, anti-Ro/La+, and APS overlap	Hydroxychloroquine (HCQ), low-dose aspirin (LDA), azathioprine (AZA), and low-dose steroids	Complement (C3/C4), anti-dsDNA, proteinuria, and fetal growth scans	Preterm birth, FGR, preeclampsia, lupus flare, and miscarriage
Antiphospholipid Syndrome (APS)	Triple aPL positivity, prior thrombosis, LA positivity, and FGR/stillbirth history	LDA + LMWH; in refractory cases, consider HCQ, low-dose prednisone, and IVIg	Fetal Doppler/echocardiography, aPL profile, and placental function	Stillbirth, preterm birth, FGR, thrombosis, and placental failure
Sjögren’s Syndrome (SS)	Anti-Ro/SSA and/or anti-La/SSB positivity, aPL positivity, and CHB history	HCQ (especially in Ro+) and corticosteroids only if indicated	Weekly fetal echocardiography (16–28 weeks) and maternal anti-Ro/La titers	CHB, miscarriage, preterm birth, SGA, and neonatal lupus
Systemic Sclerosis (SSc)	Pulmonary arterial hypertension, renal crisis history, ILD, and diffuse cutaneous disease	Nifedipine (Raynaud’s), HCQ, AZA, and LDA; avoid teratogenic agents	Cardiopulmonary evaluation, renal function, fetal growth, and BP	Preterm birth, miscarriage, low birthweight, and maternal complications
Rheumatoid Arthritis (RA)	Active disease at conception, high CRP, and chronic corticosteroid use	HCQ, sulfasalazine, AZA, and selected TNF inhibitors (certolizumab and etanercept)	Disease activity score, BP, fetal growth, and drug exposure timing	Preterm birth, miscarriage, FGR, congenital anomalies, and cesarean delivery

Abbreviations: APL—antiphospholipid antibodies; APS—antiphospholipid syndrome; AZA—azathioprine; BP—blood pressure; CHB—congenital heart block; CRP—C-reactive protein; FGR—fetal growth restriction; HCQ—hydroxychloroquine; IVIg—intravenous immunoglobulin; LA—lupus anticoagulant; LDA—low-dose aspirin; LMWH—low-molecular-weight heparin; RA—rheumatoid arthritis; SGA—small for gestational age; SLE—systemic lupus erythematosus; SSc—systemic sclerosis; SS—Sjögren’s syndrome; TNF—tumor necrosis factor.

**Table 2:** Commonly used medications in rheumatic diseases during pregnancy: typical dosages, indications, and safety profiles.

Medication	Typical Dosage During Pregnancy	Main Indications	Pregnancy Safety	Key Side Effects
Hydroxychloroquine (HCQ)	200–400 mg/day (≤6.5 mg/kg/day)	SLE, APS (adjunct), Sjögren’s syndrome, and RA	Safe in all trimesters	Retinopathy (rare) and gastrointestinal upset
Azathioprine (AZA)	≤2 mg/kg/day	SLE, RA, and other autoimmune connective tissue diseases	Safe if ≤2 mg/kg/day	Bone marrow suppression and hepatotoxicity
Low-Dose Aspirin (LDA)	75–150 mg/day	Prevention of preeclampsia; APS	Safe	Gastrointestinal bleeding and hypersensitivity
Low-Molecular-Weight Heparin (LMWH)	Prophylactic: 40 mg/day SC; Therapeutic: 1 mg/kg SC every 12 h	APS and prevention of venous thromboembolism (VTE)	Safe (does not cross placenta)	Injection-site hematoma and thrombocytopenia
Prednisone/Prednisolone	≤10–20 mg/day (short courses up to 1 mg/kg/day)	SLE flares, RA, and exacerbations of connective tissue diseases	Safe (preferably <20 mg/day)	Gestational diabetes, hypertension, and risk of preterm birth
Tacrolimus	0.05–0.1 mg/kg/day (adjusted to trough levels)	Refractory SLE, lupus nephritis (LN), and RA	Considered as safe	Nephrotoxicity, hypertension, and hyperglycemia

Abbreviations: APS—antiphospholipid syndrome; AZA—azathioprine; CTD—connective tissue disease; GI—gastrointestinal; HCQ—hydroxychloroquine; LDA—low-dose aspirin; LMWH—low-molecular-weight heparin; LN—lupus nephritis; RA—rheumatoid arthritis; SC—subcutaneous; SLE—systemic lupus erythematosus.

## Data Availability

No new data were created or analyzed in this study. Data sharing is not applicable to this article.

## Abbreviations

aCL	anticardiolipin antibodies
aPL	antiphospholipid antibodies
APS	antiphospholipid syndrome
ASA	acetylsalicylic acid
AZA	azathioprine
BP	blood pressure
CHB	congenital heart block
CI	confidence interval
CRP	C-reactive protein
CTD	connective tissue disease
DMARDs	disease-modifying antirheumatic drugs
FGR	fetal growth restriction
GI	Gastrointestinal
GWAS	genome-wide association study
HCQ	Hydroxychloroquine
IVIg	intravenous immunoglobulin
LA	lupus anticoagulant
LDA	low-dose aspirin
LMWH	low-molecular-weight heparin
LN	lupus nephritis
NC-OAPS	non-criteria obstetric antiphospholipid syndrome
OAPS	obstetric antiphospholipid syndrome
OMAPS	obstetric morbidity APS
OR	odds ratio
PAH	pulmonary arterial hypertension
PEX	plasma exchange
RA	rheumatoid arthritis
RCT	randomized controlled trial
SC	Subcutaneous
SGA	small for gestational age
SLE	systemic lupus erythematosus
SLEPDAI	systemic lupus erythematosus pregnancy disease activity index
SLICC	systemic lupus international collaborating clinics
SS	Sjögren’s syndrome
SSc	systemic sclerosis
TNF	tumor necrosis factor
UFH	unfractionated heparin
VTE	venous thromboembolism

## References

[1] Marder W., Littlejohn E.A., Somers E.C. (2016). Pregnancy and autoimmune connective tissue diseases. Best Pr. Res. Clin. Rheumatol..

[2] Nodler J., Moolamalla S.R., Ledger E.M., Nuwayhid B.S., Mulla Z.D. (2009). Elevated antiphospholipid antibody titers and adverse pregnancy outcomes: Analysis of a population-based hospital dataset. BMC Pregnancy Childbirth.

[3] Clark C.A., Spitzer K.A., Laskin C.A. (2005). Decrease in pregnancy loss rates in patients with systemic lupus erythematosus over a 40-year period. J. Rheumatol..

[4] Merz W.M., Fischer-Betz R., Hellwig K., Lamprecht G., Gembruch U. (2022). Pregnancy and autoimmune disease: Diseases of the nervous system, connective tissue, and the bowel. Dtsch. Arztebl. Int..

[5] Girardi G., Longo M., Bremer A.A. (2023). Social determinants of health in pregnant individuals from underrepresented, understudied, and underreported populations in the United States. Int. J. Equity Health.

[6] Barber M.R.W., Falasinnu T., Ramsey-Goldman R., Clarke A.E. (2023). The global epidemiology of SLE: Narrowing the knowledge gaps. Rheumatology.

[7] Khogali H.I., Al-Bluwi G.S.M., Pedo V.G., Al Dhanhani A.M. (2023). Maternal and fetal health outcomes in systemic lupus erythematosus pregnancies in the Emirati population: A comparative study. Lupus.

[8] Lu J., Xu D., Wan Q., Chen H. (2024). Pregnancy outcomes and risk factors analysis in patients with systemic lupus erythematous. BMC Pregnancy Childbirth.

[9] Bin Joo Y., Kim K.-J., Park K.-S., Park Y.-J. (2021). Pregnancy rates and perinatal outcomes in women with systemic lupus erythematosus: Data from the Korean national health claims database. Clin. Rheumatol..

[10] Tsuda S., Sameshima A., Sekine M., Kawaguchi H., Fujita D., Makino S., Morinobu A., Murakawa Y., Matsui K., Sugiyama T. (2020). Pre-conception status, obstetric outcome and use of medications during pregnancy of systemic lupus erythematosus (SLE), rheumatoid arthritis (RA) and inflammatory bowel disease (IBD) in Japan: Multi-center retrospective descriptive study. Mod. Rheumatol..

[11] Al-Riyami N., Salman B., Al-Rashdi A., Al-Dughaishi T., Al-Haddabi R., Hassan B. (2021). Pregnancy Outcomes in Systemic Lupus Erythematosus Women. Sultan Qaboos Univ. Med. J..

[12] Poh Y.J., Yii I.Y.L., Goh L.H., Li H.H., Yang L., Tan H.K., Thumboo J., Tan L.K. (2020). Maternal and Fetal Outcomes in Systemic Lupus Erythematosus Pregnancies. Ann. Acad. Med. Singap..

[13] Erazo-Martínez V., Nieto-Aristizábal I., Ojeda I., González M., Aragon C.C., Zambrano M.A., Tobón G.J., Arango J., Echeverri A., Aguirre-Valencia D. (2021). Systemic erythematosus lupus and pregnancy outcomes in a Colombian cohort. Lupus.

[14] Bremme K., Honkanen S., Gunnarsson I., Chaireti R. (2021). The presence of lupus nephritis additionally increases the risk of preeclampsia among pregnant women with systemic lupus erythematosus. Lupus.

[15] Çetin Ç., Saraç-Sivrikoz T., Ateş-Tıkız M., Zaralı S., Ersoy A., Yalçınkaya Y., Gül A., İnanç M., Has R., Kalelioğlu İ. (2023). The correlation between pregnancy, disease activity and adverse pregnancy outcomes in patients with systemic lupus erythematosus. Lupus.

[16] Braga A., Barros T., Faria R., Marinho A., Carvalheira G., Rocha G., Farinha F., Neves E., Vasconcelos C., Braga J. (2021). Systemic lupus erythematosus and pregnancy: A retrospective single-center study of 215 pregnancies from Portugal. Lupus.

[17] Mokbel A., Attia D.H., Zayed H.S., Naeem N.E., Mahmoud G., Riad R., Elewa S.A., Youssef M., Haggag H., Mohamed S.S. (2023). Pregnancy outcomes among Egyptian women with systemic lupus erythematosus: A prospective cohort study. Lupus.

[18] Chen X., Di W., Ye L., Hu Y., Jiang M., Wu J., Bu J., Sun J., Bei F. (2023). Association of preterm outcome with maternal systemic lupus erythematosus: A retrospective cohort study. Ital. J. Pediatr..

[19] Madanchi N., Fava A., Goldman D.W., Magder L.S., Petri M. (2025). Association Between 25-hydroxyvitamin D Levels and Adverse Pregnancy Outcomes in Systemic Lupus Erythematosus. Arthritis Care Res..

[20] Muñoz C.M., Goulden B., Ahmed K., Alijotas-Reig J., Giles I. (2023). Risk of adverse pregnancy outcomes prior to the onset of an autoimmune rheumatic disease: A systematic review. Rheumatology.

[21] Laíño-Piñeiro M.-C., Rúa-Figueroa I., Jiménez N., Lozano M.J.C., Martínez-Barrio J., Serrano B., Galindo-Izquierdo M., Nack A., Loricera J., Tomero-Muriel E. (2023). Pregnancy outcomes in 1869 pregnancies in a large cohort from the Spanish Society of Rheumatology Lupus Register (RELESSER). Semin. Arthritis Rheum..

[22] Wind M., Fierro J.J., Bloemenkamp K.W.M., de Leeuw K., Lely A.T., Limper M., Sueters M., Teng Y.K.O., Walter I.J., Kooiman J. (2024). Pregnancy outcome predictors in systemic lupus erythematosus: A systematic review and meta-analysis. Lancet Rheumatol..

[23] Crisafulli F., Andreoli L., Zucchi D., Reggia R., Gerardi M.C., Lini D., Tani C., Zatti S., Franceschini F., Mosca M. (2023). Variations of C3 and C4 Before and During Pregnancy in Systemic Lupus Erythematosus: Association with Disease Flares and Obstetric Outcomes. J. Rheumatol..

[24] Hiramatsu Y., Isoda K., Kotani T., Nakamura E., Wada Y., Fujiki Y., Makino S., Fujita D., Takeuchi T. (2021). Pre-pregnancy serum complement C3 level is a predictor of preterm birth for pregnancies with systemic lupus erythematosus. Arthritis Res. Ther..

[25] Radin M., Cecchi I., Crisafulli F., Klumb E.M., de Jesús G.R., Lacerda M.I., Saavedra M.Á., Reyes-Navarro G.V., Iaccarino L., Larosa M. (2023). Complement levels during the first trimester predict disease flare and adverse pregnancy outcomes in systemic lupus erythematosus: A network meta-analysis on 532 pregnancies. Autoimmun. Rev..

[26] Ong S.G., Ding H.J. (2021). Predictors of adverse pregnancy outcome in a cohort of women with systemic lupus erythematosus in Malaysia. Med. J. Malays..

[27] Zucchi D., Fischer-Betz R., Tani C. (2023). Pregnancy in systemic lupus erythematosus. Best Pr. Res. Clin. Rheumatol..

[28] Huang J., Zhu Q., Wang B., Wang H., Xie Z., Zhu X., Zhao T., Yang Z. (2024). Antiphospholipid antibodies and the risk of adverse pregnancy outcomes in patients with systemic lupus erythematosus: A systematic review and meta-analysis. Expert Rev. Clin. Immunol..

[29] Zamani B., Shayestehpour M., Esfahanian F., Akbari H. (2020). The study of factors associated with pregnancy outcomes in patients with systemic lupus erythematosus. BMC Res. Notes.

[30] Ravindran V., Bhadran S., Divakaran M., Reshma V.M. (2024). Lupus pregnancy outcomes in women with previous adverse outcomes: A prospective cohort study. Clin. Rheumatol..

[31] Janardana R., Haridas V., Priya V., Bhat V., Singh Y., Rao V.K., Jois R., Srikantiah C., Pinto B., Shobha V. (2020). Maternal and fetal outcomes of lupus pregnancies: A collective effort by Karnataka Rheumatologists. Lupus.

[32] Duan J., Ma D., Wen X., Guo Q., Gao J., Zhang G., Xu K., Zhang L. (2021). Hydroxychloroquine prophylaxis for preeclampsia, hypertension and prematurity in pregnant patients with systemic lupus erythematosus: A meta-analysis. Lupus.

[33] Clowse M.E.B., Eudy A.M., Balevic S., Sanders-Schmidler G., Kosinski A., Fischer-Betz R., Gladman D.D., Molad Y., Nalli C., Mokbel A. (2022). Hydroxychloroquine in the pregnancies of women with lupus: A meta-analysis of individual participant data. Lupus Sci. Med..

[34] Do S.C., Rizk N.M., Druzin M.L., Simard J.F. (2020). Does Hydroxychloroquine Protect Against Preeclampsia and Preterm Delivery in Systemic Lupus Erythematosus Pregnancies?. Am. J. Perinatol..

[35] Hu Z., Gao R., Huang W., Wang H., Qin L. (2023). Effect of Hydroxychloroquine on Lupus Activity, Preeclampsia and Intrauterine Growth Restriction in Pregnant Women with Systemic Lupus Erythematosus and/or Antiphospholipid Syndrome: A Systematic Review and Meta-Analysis. J. Clin. Med..

[36] Shimada H., Wakiya R., Kanenishi K., Miyatake N., Nakashima S., Mansour M.M.F., Kato M., Miyagi T., Sugihara K., Ushio Y. (2022). Preterm birth is strongly affected by the glucocorticoid dose during pregnancy in women complicated by systemic lupus erythematosus. Arthritis Res. Ther..

[37] Louthrenoo W., Trongkamolthum T., Kasitanon N., Wongthanee A. (2021). Predicting factors of adverse pregnancy outcomes in Thai patients with systemic lupus erythematosus. Medicine.

[38] Irino K., Arinobu Y., Ayano M., Kawano S., Kimoto Y., Mitoma H., Akahoshi M., Akashi K., Horiuchi T., Niiro H. (2021). Predictive factors of fetal and maternal pregnancy outcomes in Japanese patients with systemic lupus erythematosus: A STROBE-compliant study. Lupus.

[39] Tani C., Zucchi D., Haase I., Gerosa M., Larosa M., Cavagna L., Bortoluzzi A., Crisafulli F., Mucke J., Strigini F.A.L. (2022). Impact of low-dose acetylsalicylic acid on pregnancy outcome in systemic lupus erythematosus: Results from a multicentre study. Lupus Sci. Med..

[40] Nakai T., Kitada A., Fukui S., Okada M. (2021). Risk of adverse pregnancy outcomes in Japanese systemic lupus erythematosus patients with prior severe organ manifestations: A single-center retrospective analysis. Lupus.

[41] Fierro J.J., Prins J.R., Verstappen G.M., Bootsma H., Westra J., de Leeuw K. (2023). Preconception clinical factors related to adverse pregnancy outcomes in patients with systemic lupus erythematosus or primary Sjögren’s syndrome: A retrospective cohort study. RMD Open.

[42] Dabit J.Y., Valenzuela-Almada M.O., Vallejo-Ramos S., Duarte-García A. (2022). Epidemiology of Antiphospholipid Syndrome in the General Population. Curr. Rheumatol. Rep..

[43] Alijotas-Reig J., Esteve-Valverde E., Ferrer-Oliveras R., Sáez-Comet L., Lefkou E., Mekinian A., Belizna C., Ruffatti A., Hoxha A., Tincani A. (2021). Comparative study of obstetric antiphospholipid syndrome (OAPS) and non-criteria obstetric APS (NC-OAPS): Report of 1640 cases from the EUROAPS registry. Rheumatology.

[44] D’ippolito S., Barbaro G., Paciullo C., Tersigni C., Scambia G., Di Simone N. (2023). Antiphospholipid Syndrome in Pregnancy: New and Old Pathogenetic Mechanisms. Int. J. Mol. Sci..

[45] Mineo C., Shaul P.W., Bermas B.L. (2023). The pathogenesis of obstetric APS: A 2023 update. Clin. Immunol..

[46] Xu J., Chen D., Tian Y., Wang X., Peng B., Athalye-Jape G.K. (2022). Antiphospholipid Antibodies Increase the Risk of Fetal Growth Restriction: A Systematic Meta-Analysis. Int. J. Clin. Pr..

[47] Iordache O., Anastasiu D.M., Kakarla M., Ali A., Bratosin F., Neamtu R., Dumitru C., Olaru F., Erdelean I., Gherman A. (2023). Influence of Antiphospholipid Antibody-Associated Thrombophilia on the Risk of Preterm Birth: A Systematic Review. J. Clin. Med..

[48] Xie A., Jin Z., Li C., Li C., Luo G., Zhang X., Jian S., Li D., Xie Y., Xie L. (2025). Antiphospholipid syndrome in patients with fetal death: A prospective longitudinal cohort study. Clin. Exp. Med..

[49] Jin J., Zhang M., Cai X., Hou Y., Xiang X., Hou L., Li J., Li C. (2025). Severe thrombocytopenia is associated with adverse pregnancy outcomes in patients with obstetric antiphospholipid syndrome. Eur. J. Med Res..

[50] Walter I.J., Haneveld M.J.K., Lely A.T., Bloemenkamp K.W.M., Limper M., Kooiman J. (2021). Pregnancy outcome predictors in antiphospholipid syndrome: A systematic review and meta-analysis. Autoimmun. Rev..

[51] Tanimura K., Saito S., Nakatsuka M., Nagamatsu T., Fujii T., Fukui A., Deguchi M., Sasagawa Y., Arase N., Arase H. (2020). The β_2_-Glycoprotein I/HLA–DR Complex as a Major Autoantibody Target in Obstetric Antiphospholipid Syndrome. Arthritis Rheumatol..

[52] Matraszek V.V., Krofta L., Hromadnikova I. (2025). Even low levels of anticardiolipin antibodies are associated with pregnancy-related complications: A monocentric cohort study. Acta Obstet. Gynecol. Scand..

[53] Murarasu A., Guettrot-Imbert G., Le Guern V., Yelnik C., Queyrel V., Schleinitz N., Ferreira-Maldent N., Diot E., Urbanski G., Pannier E. (2022). Characterisation of a high-risk profile for maternal thrombotic and severe haemorrhagic complications in pregnant women with antiphospholipid syndrome in France (GR2): A multicentre, prospective, observational study. Lancet Rheumatol..

[54] Wang B., Wang Q., Yu D., Zhang N., Wang Z., Sun X., Liu M., Su X. (2025). Using Doppler ultrasound to assess fetal cardiac function and pregnancy outcomes in obstetric antiphospholipid syndrome pregnancies: A case–control study. Arch. Gynecol. Obstet..

[55] Moyle K.A., Branch D.W., Peterson L.K., Guerra M.M., Allshouse A.A., Benson A.E., Salmon J.E. (2024). Association Between Novel Antiphospholipid Antibodies and Adverse Pregnancy Outcomes. Obstet. Gynecol..

[56] Yang Z., Shen X., Zhou C., Wang M., Liu Y., Zhou L. (2021). Prevention of recurrent miscarriage in women with antiphospholipid syndrome: A systematic review and network meta-analysis. Lupus.

[57] Frishman M., Radin M., Schreiber K. (2020). Hydroxychloroquine and antiphospholipid antibody-related pregnancy morbidity: A systematic review and meta-analysis. Curr. Opin. Obstet. Gynecol..

[58] Khizroeva J., Bitsadze V., Tincani A., Makatsariya A., Arslanbekova M., Babaeva N., Tsibizova V., Shkoda A., Makatsariya N., Tretyakova M. (2022). Hydroxychloroquine in obstetric antiphospholipid syndrome: Rationale and results of an observational study of refractory cases. J. Matern. Neonatal Med..

[59] Hooper A., Bacal V., Bedaiwy M.A. (2023). Does adding hydroxychloroquine to empiric treatment improve the live birth rate in refractory obstetrical antiphospholipid syndrome? A systematic review. Am. J. Reprod. Immunol..

[60] Gerde M., Ibarra E., Mac Kenzie R., Suarez C.F., Heer C., Alvarez R., Iglesias M., Balparda J., Beruti E., Rubinstein F. (2021). The impact of hydroxychloroquine on obstetric outcomes in refractory obstetric antiphospholipid syndrome. Thromb. Res..

[61] Riancho-Zarrabeitia L., Lopez-Marin L., Cacho P.M., López-Hoyos M., del Barrio R., Haya A., Martínez-Taboada V.M. (2022). Treatment with low-dose prednisone in refractory obstetric antiphospholipid syndrome: A retrospective cohort study and meta-analysis. Lupus.

[62] Urban M.L., Bettiol A., Serena C., Comito C., Turrini I., Fruttuoso S., Silvestri E., Vannacci A., Ravaldi C., Petraglia F. (2020). Intravenous immunoglobulin for the secondary prevention of stillbirth in obstetric antiphospholipid syndrome: A case series and systematic review of literature. Autoimmun. Rev..

[63] Kaneko K., Tsutsumi S., Fujita D., Sugiura-Ogasawara M., Mitsuda N., Matsubara K., Atsumi T., Inoue E., Takimoto T., Murashima A. (2024). Intravenous immunoglobulin treatment for obstetric antiphospholipid syndrome refractory to conventional therapy: A single-arm, open-labelled multicentre clinical trial. Mod. Rheumatol..

[64] Hoxha A., Tormene D., Campello E., Simioni P. (2022). Treatment of Refractory/High-Risk Pregnancies with Antiphospholipid Syndrome: A Systematic Review of the Literature. Front. Pharmacol..

[65] Ruffatti A., Tonello M., Favaro M., Del Ross T., Calligaro A., Ruffatti A.T., Gervasi M.T., Hoxha A. (2021). The efficacy and safety of second-line treatments of refractory and/or high risk pregnant antiphospholipid syndrome patients. A systematic literature review analyzing 313 pregnancies. Semin. Arthritis Rheum..

[66] Geng B., Zhang K., Huang X., Chen Y. (2022). A meta-analysis of the effect of Sjögren′s syndrome on adverse pregnancy outcomes. Clinics.

[67] Maleki-Fischbach M., Kastsianok L., Koslow M., Chan E.D. (2024). Manifestations and management of Sjögren’s disease. Arthritis Res. Ther..

[68] Yang Y., Huang X.-X., Huo R.-X., Lin J.-Y. (2024). Impact of Sjögren’s syndrome on maternal and fetal outcomes following pregnancy: A systematic review and meta-analysis of studies published between years 2007–2022. Arch. Gynecol. Obstet..

[69] Oliveira F.R., Valim V., Pasoto S.G., Fernandes M.L.M.S., Lopes M.L.L., Fialho S.C.d.M.S., Pinheiro A.C., dos Santos L.C., Appenzeller S., Fidelix T. (2021). 2021 recommendations of the Brazilian Society of Rheumatology for the gynecological and obstetric care of patients with Sjogren’s syndrome. Adv. Rheumatol..

[70] Fernández-Buhigas I. (2022). Obstetric management of the most common autoimmune diseases: A narrative review. Front. Glob. Women’s Health.

[71] Kaizer A.M., Lindblade C., Clancy R., Tebo A.E., Drewes B., Masson M., Chang M., Fraser N., Buyon J.P., Cuneo B.F. (2022). Reducing the burden of surveillance in pregnant women with no history of fetal atrioventricular block using the negative predictive value of anti-Ro/SSA antibody titers. Am. J. Obstet. Gynecol..

[72] Bairkdar M., Rossides M., Westerlind H., Hesselstrand R., Arkema E.V., Holmqvist M. (2021). Incidence and prevalence of systemic sclerosis globally: A comprehensive systematic review and meta-analysis. Rheumatology.

[73] Lazzaroni M.-G., Crisafulli F., Moschetti L., Semeraro P., Cunha A.-R., Neto A., Lojacono A., Ramazzotto F., Zanardini C., Zatti S. (2023). Reproductive Issues and Pregnancy Implications in Systemic Sclerosis. Clin. Rev. Allergy Immunol..

[74] Chung M.P., Kolstad K.D., Dontsi M., Postlethwaite D., Manwani P., Zhao H., Kesh S., Simard J.F., Chung L. (2022). Increased Rates of Obstetric Complications Prior to Systemic Sclerosis Diagnosis. Arthritis Care Res..

[75] Braun J., Balbir-Gurman A., Toledano K., Tavor Y., Braun-Moscovici Y. (2022). Favourable outcome of planned pregnancies in systemic sclerosis patients during stable disease. Scand. J. Rheumatol..

[76] Sammaritano L.R., Bermas B.L., Chakravarty E.E., Chambers C., Clowse M.E.B., Lockshin M.D., Marder W., Guyatt G., Branch D.W., Buyon J. (2020). 2020 American College of Rheumatology Guideline for the Management of Reproductive Health in Rheumatic and Musculoskeletal Diseases. Arthritis Care Res..

[77] Russell M.D., Dey M., Flint J., Davie P., Allen A., Crossley A., Frishman M., Gayed M., Hodson K., Khamashta M. (2023). British Society for Rheumatology guideline on prescribing drugs in pregnancy and breastfeeding: Immunomodulatory anti-rheumatic drugs and corticosteroids. Rheumatology.

[78] Neuman R.I., Smeele H.T.W., Danser A.H.J., Dolhain R.J.E.M., Visser W. (2022). The sFlt-1 to PlGF ratio in pregnant women with rheumatoid arthritis: Impact of disease activity and sulfasalazine use. Rheumatology.

[79] Venetsanopoulou A.I., Alamanos Y., Voulgari P.V., Drosos A.A. (2023). Epidemiology and Risk Factors for Rheumatoid Arthritis Development. Mediterr. J. Rheumatol..

[80] Huang W., Wu T., Jin T., Zhang Y., Wang J., Qi J., Li Y., Jiang H., Zhang J., Jiang Z. (2023). Maternal and fetal outcomes in pregnant women with rheumatoid arthritis: A systematic review and meta-analysis. Clin. Rheumatol..

[81] Sim B.L.B., Daniel R.S.B., Hong S.S.B., Matar R.H.B., Ganiel I.B., Nakanishi H.M., Mansour R.B., Than C.A., Alrahmani L. (2023). Pregnancy Outcomes in Women with Rheumatoid Arthritis. Am. J. Clin. Oncol..

[82] Lv J., Xu L., Mao S. (2023). Association between disease activity of rheumatoid arthritis and maternal and fetal outcomes in pregnant women: A systematic review and meta-analysis. BMC Pregnancy Childbirth.

[83] Zhang D., Hu Y., Guo W., Song Y., Yang L., Yang S., Ou T., Liu Y., Zhang Y. (2022). Mendelian randomization study reveals a causal relationship between rheumatoid arthritis and risk for pre-eclampsia. Front. Immunol..

[84] Tian L.M., Zhang Z.M., Mao Y.M., Zong M. (2023). Association between pregnant women with rheumatoid arthritis and preeclampsia: A systematic review and meta-analysis. Medicine.

[85] Knudsen S., Thomsen A., Deleuran B., Bech B. (2021). Maternal rheumatoid arthritis during pregnancy and neurodevelopmental disorders in offspring: A systematic review. Scand. J. Rheumatol..

[86] Saulescu I.C., Panaitescu A.M., Gică N., Grădinaru E., Opris-Belinski D. (2024). Pre-Pregnancy Counselling for Women with Rheumatoid Arthritis: A Guide on Risks, Evaluations, and Multidisciplinary Approaches. J. Clin. Med..

[87] Peterson E.A.P., Lynton J.P., Bernard A.P., Santillan M.K., Bettendorf B. (2020). Rheumatologic Medication Use During Pregnancy. Obstet. Gynecol..

[88] Siegel C.H., Sammaritano L.R. (2024). Safety of Medications Used to Treat Autoimmune Rheumatic Diseases During Pregnancy and Lactation. Am. J. Clin. Oncol..

[89] Barenbrug L., Groen M.T., Hoentjen F., van Drongelen J., van den Reek J.M., Joosten I., de Jong E.M., van der Molen R.G. (2021). Pregnancy and neonatal outcomes in women with immune mediated inflammatory diseases exposed to anti-tumor necrosis factor-α during pregnancy: A systemic review and meta-analysis. J. Autoimmun..

[90] Nguyen H., Ahmed K., Luo W., Flint J., Giles I. (2021). A Systematic Review of the safety of non-TNF inhibitor biologic and targeted synthetic drugs in rheumatic disease in pregnancy. Semin. Arthritis Rheum..

[91] Raine C., Austin K., Giles I. (2020). Mechanisms determining the amelioration of rheumatoid arthritis in pregnancy: A systematic review. Semin. Arthritis Rheum..

[92] Mouyis M. (2020). Postnatal Care of Woman with Rheumatic Diseases. Adv. Ther..

[93] Skorpen C.G., Hoeltzenbein M., Tincani A., Fischer-Betz R., Elefant E., Chambers C., da Silva J., Nelson-Piercy C., Cetin I., Costedoat-Chalumeau N. (2016). The EULAR points to consider for use of antirheumatic drugs before pregnancy, and during pregnancy and lactation. Ann. Rheum. Dis..

[94] Taylor P.C., Weinblatt M.E., McInnes I.B., Atsumi T., Strand V., Takeuchi T., Bracher M., Brooks D., Davies J., Goode C. (2023). Anti-GM-CSF otilimab versus sarilumab or placebo in patients with rheumatoid arthritis and inadequate response to targeted therapies: A phase III randomised trial (contRAst 3). Ann. Rheum. Dis..

[95] Kim C.Y.P., Suh C.H. (2021). The safety of medications during pregnancy and lactation in patients with inflammatory rheumatic diseases. EMJ Rheumatol..

[96] Mysler E., Monticielo O.A., Al-Homood I.A., Lau C.S., Hussein H., Chen Y.-H. (2024). Opportunities and challenges of lupus care in Latin America, the Middle East, and Asia-Pacific: A call to action. Mod. Rheumatol..

[97] Flower C. (2021). Clinical Images: Diffuse systemic sclerosis, skin bleaching, and telangiectasia. Arthritis Rheumatol..

[98] Shen G., Swaminathan M., Huang I., Louden D., Feterman D., Tahir M.W., Singh N. (2023). Racial disparities in pregnancy outcomes among women with rheumatic diseases: A systematic literature review. Semin. Arthritis Rheum..

[99] Hassan M., Awan F.M., Naz A., Deandrés-Galiana E.J., Alvarez O., Cernea A., Fernández-Brillet L., Fernández-Martínez J.L., Kloczkowski A. (2022). Innovations in Genomics and Big Data Analytics for Personalized Medicine and Health Care: A Review. Int. J. Mol. Sci..

